# Deformation Analysis of Reinforced Beams Made of Lightweight Aggregate Concrete

**DOI:** 10.3390/ma13010020

**Published:** 2019-12-19

**Authors:** Darius Bacinskas, Deividas Rumsys, Aleksandr Sokolov, Gintaris Kaklauskas

**Affiliations:** 1 Department of Reinforced Concrete Structures and Geotechnics, Vilnius Gediminas Technical University, Sauletekio al. 11, LT-10223 Vilnius, Lithuania; gintaris.kaklauskas@vgtu.lt; 2 SRP Projektas Ltd., Savanorių pr. 176c, LT-03154 Vilnius, Lithuania; deividas.rumsys@srp-projektas.lt; 3 Laboratory of Innovative Building Structures, Vilnius Gediminas Technical University, Sauletekio av. 11, LT-10223 Vilnius, Lithuania; aleksandr.sokolov@vgtu.lt

**Keywords:** lightweight aggregate concrete, reinforced concrete, flexural elements, curvature, short-term loading, tension stiffening, constitutive model, numerical modeling

## Abstract

In the present trend of constructing taller and longer structures, the application of lightweight aggregate concrete is becoming an increasingly important advanced solution in the modern construction industry. In engineering practice, the analysis of lightweight concrete elements is performed using the same algorithms that are applied for normal concrete elements. As an alternative to traditional engineering methods, nonlinear numerical algorithms based on constitutive material models may be used. The paper presents a comparative analysis of curvature calculations for flexural lightweight concrete elements, incorporating analytical code methods EN 1992-1 and ACI 318-19, as well as a numerical analysis using the constitutive model of cracked tensile lightweight concrete recently proposed by the authors. To evaluate the adequacy of the theoretical predictions, experimental data of 51 lightweight concrete beams of five different programs reported in the literature were collected. A comparison of theoretical and experimental results showed that the most accurate predictions are obtained using numerical analysis and the constitutive model proposed by the authors. In the future, the latter algorithm can be used as a reliable tool for improving the design standard methods or numerical modeling of lightweight concrete elements subjected to short-term loading.

## 1. Introduction

Concrete has become the most widely used construction material worldwide. Moreover, concrete is the most widely used synthetic material. Compared with other materials, only water is used in greater quantities. Over the past 30 years, concrete production has increased by a factor of >3 times to approximately 3.8 billion m^3^ per year [1]. This represents >1 m^3^ per person per year worldwide [2]. Compared with other traditional construction materials (e.g., steel, timber, polymers, and aluminum), the amount of concrete production is twice that of other traditional materials combined. By 2050, world concrete production is projected to be a factor of four higher than the 1990 level [3].

The increasing amount of concrete production leads to a rising demand for innovative solutions for concrete structures and their implementation in real construction projects [4]. In the present trend of constructing taller and longer structures, the application of lightweight aggregate concrete is becoming an increasingly important advanced solution in the modern construction industry. Numerous studies around the world have been dedicated to research in the field of lightweight aggregate concrete. Consequently, various concrete mixtures with different mechanical properties have been proposed [5,6,7,8,9,10]. However, in most cases, traditional studies usually address the optimization of concrete properties with respect to one or more aspects, such as microstructure, mechanical resistance, and durability [6,7,8,9]. Consequently, the obtained findings do not lead to the final expected effect [5]. The improvement of selected material properties is accompanied by changes in other important parameters [9]. Moreover, experimental results are usually achieved by testing on small-scale specimens. Despite the fact that standardized techniques for material testing are usually applied, the obtained results and material models sometimes do not reflect the real mechanical behavior of large-scale load-bearing structures [11,12]. The application of advanced concrete mixes for structural members must be analyzed in an integral way—starting from the optimal composition test and ending with the evaluation of structural behavior of large-scale prototype members subjected to real operating conditions and external mechanical loading [5].

In current engineering practice, for the limit state analysis of reinforced lightweight concrete and normal concrete elements, the same algorithms are applied [13,14]. The influence of lightweight aggregate concrete on the structural behavior is taken into account by introducing additional empirical coefficients that depend on the concrete density. However, the obtained predictions of lightweight concrete members in most cases do not correspond to their real mechanical behavior—both crack width and deformation of lightweight concrete elements are underestimated [15], and the errors can reach 100%, particularly for the lightly reinforced concrete members [5]. These tendencies can be explained by the fact that lightweight concrete significantly differs from normal concrete. In particular, the properties of lightweight concrete are highly dependent on the type, amount, and mechanical properties of the selected lightweight aggregates [10,16] as well as the technological aspects of concrete mix preparation [17,18]. Traditional engineering methods, which during many years have been developed to improve normal-weight concrete mixes, are usually insufficient for evaluating these factors.

Another important, though often neglected, aspect of the serviceability analysis can be attributed to the assessment of the restrained shrinkage-induced stress–strain state at the pre-loading stage [19,20,21,22]. Some researchers [14,21,23,24] note that early-age cracking of reinforced lightweight concrete elements occurs, in particular, because of shrinkage of concrete in combination with lower tensile strength. These effects are not taken into account in traditional engineering techniques.

The application of performance-based design concepts in advanced structural engineering has increased the integration of alternative numerical methods in the design process of complex modern structures [22,25]. Adequate constitutive models representing the behavior of concrete and reinforcement, as well as their interaction, must be used in the following algorithms [26,27]. Numerous physical models have been proposed for the analysis of conventional reinforced concrete elements [19,28,29,30]. However, studies in the field of constitutive modeling of lightweight aggregate concrete are insufficient and still require a solution because advanced lightweight concrete is a relatively new material [31].

This paper presents a comparative analysis of curvature calculations for flexural lightweight aggregate concrete elements, incorporating analytical code methods (EN 1992-1 (EC2) [32] and ACI 318-19 (ACI) [33]), as well as a numerical analysis using the constitutive model of cracked tensile lightweight concrete recently proposed by the authors [5,23]. To evaluate the adequacy of the theoretical predictions, experimental data of 51 lightweight concrete beams tested during five different programs were collected. The reinforcement ratio of the experimental beams ranged from 0.33% to 2.82%, the density ranged from 1651 to 2000 kg/m^3^, and the compressive strength of concrete ranged from 20 to 70 MPa. A comparison of theoretical and experimental results showed that the most accurate predictions are obtained using numerical analysis and the constitutive model proposed by the authors. In the future, the latter algorithm can be used as a reliable tool for improving the design standard methods or numerical modeling of lightweight concrete elements subjected to short-term loading.

## 2. Calculation Methods Employed for Comparative Analysis

### 2.1. Eurocode 2 (EC2)

According to EC2 [32] methodology, curvatures of reinforced lightweight concrete beams are calculated using the same relationships as for traditional reinforced concrete elements. The algorithm distinguishes two stages of deformation of reinforced concrete elements. In the first stage (before cracking), the element behavior is fully elastic, and the curvature is calculated by applying the fundamental relationships of material mechanics. In the second stage (during which the element is fully cracked), tensile stresses are entirely carried by the tensile reinforcement. At this stage, the curvature is calculated using the geometric characteristics of the fully cracked cross section. 

The mean curvature at any intermediate stress–strain stage can be assessed by interpolation between values calculated for stages I and II. Using this concept, the tension-stiffening effect is taken into account. The mean curve is calculated by the following formula:

(1) $$\kappa =\left(1-\zeta \right)\frac{M}{E_{lcm}I_{u}}+\zeta \frac{M}{E_{lcm}I_{c}},$$

where *κ* is the mean curvature of the cross section, *M* is the bending moment at the considered load level, *I_u_* is the moment of inertia of the non-cracked cross section, and *I_c_* is the moment of inertia of the fully cracked cross section. *E_lcm_* is the average modulus of elasticity of lightweight aggregate concrete calculated by using

(2) $$E_{lcm}=22{\left(f_{lcm}/10\right)}^{0.3}{\left(\rho /2200\right)}^{2},$$

where *f_lcm_* is the average compressive strength of lightweight aggregate concrete, and *ρ* is the density of concrete. Here, *ζ* is the interpolation coefficient; if the cross section is not cracked, *ζ* = 0; otherwise, it is calculated by using

(3) $$\zeta =1-\beta {\left(\frac{M_{cr}}{M}\right)}^{2},$$

where *M_cr_* is the cracking moment, and *β* is the coefficient that takes into account the influence of the loading duration (short or long-term) as well as type of loading (static or cyclic) on the average deformations. The coefficient *β* is 1 and 0.5 for short-term static loads and long-term or cyclical loads, respectively.

EC2 also provides an expression for the calculation of curvature *κ_cs_* caused by concrete shrinkage deformations:

(4) $$\kappa_{cs}=\varepsilon_{cs}\alpha_{e}\frac{S}{I},$$

where *ε_cs_* is the free shrinkage deformation, *α_e_* is the ratio of reinforcement and concrete modulus of elasticity (effective modular ratio), *S* is the first moment of area of the reinforcement about the centroid of the section, and *I* is the second moment of the area of the section. The above relationship is commonly used to calculate long-term curvatures with curvature increases caused by shrinkage taken into account. However, EC2 does not provide any direct recommendations for short-term deformational analysis to evaluate the shrinkage effect in the pre-loading stage. As mentioned above, concrete free shrinkage is restrained by reinforcement, causing tension stresses in concrete even before loading. Depending on the shrinkage value and reinforcement ratio, this can significantly decrease the cracking limit and can result in considerable prediction errors [20,21].

### 2.2. ACI 318-19 (ACI)

According to the ACI standard [33], the curvature of non-cracked cross-sectional elements is calculated using the following fundamental formula, considering elastic geometric and physical characteristics:

(5) $$\kappa =\frac{M}{E_{lc}I_{g}},$$

where *M* is the maximum bending moment, *I_g_* is the moment of inertia of the non-cracked gross section, and *E_lc_* is the modulus of elasticity of lightweight aggregate concrete calculated by the following formula:

(6) $$E_{lc}=0.043\rho^{1.5}\sqrt{f_{c}},$$

where *f_c_* is the compressive strength (in MPa).

The effective moment of inertia of the cracked cross section is calculated by interpolation between the moments of inertia of the non-cracked (*I_g_*) and the fully cracked (*I_cr_*) cross sections:

(7) $$I_{e}={\left(\frac{M_{cr}}{M}\right)}^{3}I_{g}+\left[1-{\left(\frac{M_{cr}}{M}\right)}^{3}\right]I_{cr}\leq I_{g},$$

where *M_cr_* is the cracking moment calculated as follows:

(8) $$M_{cr}=\frac{f_{r}I_{g}}{y_{t}},$$

where *f_r_* is the modulus of rupture, and *y_t_* is the distance from the centroid of the gross concrete section to the bottom tensile layer.

The curvature of the cracked element is calculated with Formula (5) using the effective moment of inertia:

(9) $$\kappa =\frac{M}{E_{lc}I_{e}}.$$

### 2.3. Numerical Method for Deformation Analysis Using a Tension-Stiffening Model of Lightweight Aggregate Concrete

**Figure 1:**
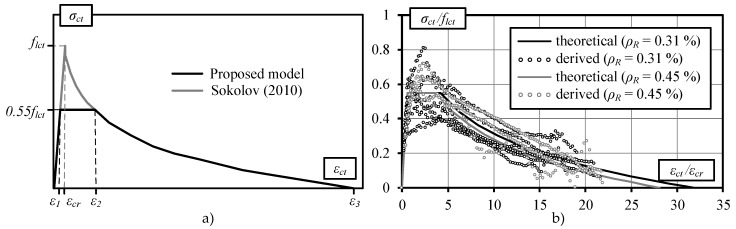
Tension–stiffening model of structural lightweight concrete: (**a**) theoretical diagrams and (**b**) normalized stress–strain diagrams obtained for the selected experimental beams [5].

As an alternative to design codes, numerical methods with incorporated constitutive models of materials can be used to assess nonlinear stress–strain behavior of reinforced concrete members. The current study applies the modified tension-stiffening relationship originally proposed by Sokolov [29] for traditional reinforced concrete. The modified and original models are presented in Figure 1a.

Constitutive modeling techniques for deriving the modified model including applied experimental results of flexural reinforced lightweight concrete beams are discussed in more detail in references [5,23]. The basic aspects of the physical modeling are presented below. The methodology is based on the layered section model, implying the successive application of the direct (curvature prediction) and inverse (constitutive modeling) approaches. The method proposed by Kaklauskas and Ghaboussi [34] was applied for constitutive modeling to obtain average stress and average strain diagrams for cracked tensile concrete. The mathematical algorithm of the applied inverse procedure is discussed in more detail in [20,29,35]. Experimental stress–strain diagrams representing the tension-stiffening effect for flexural members have been obtained by performing a three-step computation. The latter includes the elimination of the concrete shrinkage effect on the stress–strain behavior of reinforced concrete members before loading [20]. In the first step, using an inverse procedure [34], average tensile stress–strain diagrams are obtained from experimental moment–curvature relationships. In the second step, the obtained curves are used in the direct approach with shrinkage deformations taken into account. By using the above technique, the modified moment-curvature diagrams for experimental specimens are obtained by eliminating the influence of shrinkage deformations. In the final step, the modified moment–curvature diagrams are used again in the inverse algorithm. Consequently, tension–stiffening diagrams with shrinkage eliminated are derived. Examples of the normalized tension–stiffening diagrams obtained using the above procedure together with the proposed model are presented in Figure 1b. This figure represents stress–strain relations of nine experimental concrete beams reported in [5]. The beams were made of lightweight aggregate concrete with density *ρ* = 1897–1959 kg/m^3^ and were having reinforcement ratio *ρ_R_* = 0.31% and 0.45%. Stress–strain relations were derived from the test moment-curvature diagrams.

The proposed modified constitutive model (Figure 1a) is approximated by a three-curve diagram. The ascending branch of the curve represents the elastic behavior of the reinforced concrete before cracking. Meanwhile, the horizontal line and descending branch describe the stages of crack formation and further development, respectively. According to [5], the ultimate tensile strength is *σ_ct_* = 0.55*f_lct_*, where *f_lct_* is the average tensile strength of lightweight aggregate concrete calculated according to the EC2 standard:

(10) $$f_{lctm}=f_{ctm}\eta_{1},$$

(11) $$\eta_{1}=0.40+0.60\rho /2200,$$

where *ρ* is the density of lightweight aggregate concrete.

(12) $$f_{ctm}=0.30f_{lck}^{2/3}\;\mathrm{for}\;\mathrm{concrete}\;\mathrm{grade}\leq LC50/55.$$

(13) $$f_{ctm}=2.12ln\left[1+\left(\frac{f_{lck}+8}{10}\right)\right]\;\mathrm{for}\;\mathrm{concrete}\;\mathrm{grade}>LC50/55.$$

The strain *ε*_1_ corresponding to the ultimate tensile stress is determined by the following relationship:

(14) $$\varepsilon_{1}=0.55\varepsilon_{cr},$$

where *ε_cr_* = *f_lct_*/*E_lcm_* is the theoretical cracking strain corresponding to the tensile strength, and *E_lcm_* is the modulus of elasticity of concrete calculated according to EC2 depending on the compressive strength of concrete.

The shape of the descending part of the diagram is described by the following formula [5,29]:

(15) $$\sigma_{ct}=f_{lct}\left(1-0.27\ln\left(\frac{\varepsilon_{ct}}{\varepsilon_{cr}}\right)-0.21\rho_{R}\right),$$

where *ρ_R_* is the reinforcement ratio [%].

The strain *ε*_2_ is calculated by using the relationship derived in Equation (15), and the ultimate tensile stress of concrete, *σ_ct_* = 0.55*f_lct_*:

(16) $$\varepsilon_{2}=\varepsilon_{cr}e^{1.667-0.78\rho_{R}}.$$

The length of the descending branch is defined by the maximal strain *ε*_3_ corresponding to zero stress. This strain is calculated by the following formula:

(17) $$\varepsilon_{3}=\varepsilon_{cr}e^{3.7-0.78\rho_{R}}.$$

The stress–strain relations of the above constitutive law are shown in Figure 1b along with the stress–strain diagrams obtained from the experimental moment–curvature response. The comparison shows close agreement of the proposed model and the curves obtained from the tests.

**Figure 2:**
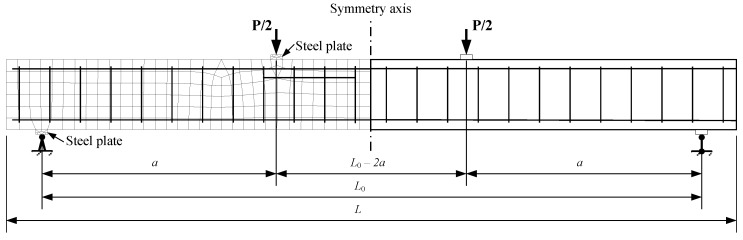
A typical finite element model and loading and support conditions of the test beams.

A nonlinear numerical analysis was performed using the finite element software ATENA (Cervenka Consulting Ltd., Prague, Czech Republic). Two-dimensional finite element models of experimental reinforced concrete elements were created employing constitutive models for compressive and tensile concrete and reinforcement. The behavior of the reinforcement is represented by an elastic–plastic model corresponding to the yield strength of steel and modulus of elasticity. A linear elastic diagram was used to model the compressive concrete. The proposed constitutive model (Figure 1a) was used to describe the behavior of lightweight aggregate concrete in tension. The 3D Non Linear Cementitious 2 User material model (based on SBETA material model offered by ATENA) was utilized. Concrete without cracks is considered as isotropic and concrete with cracks as orthotropic body. Smeared crack and fracture mechanics approaches are combined in ATENA to assess the nonlinear behavior of reinforced concrete elements after cracking. In this study, the fixed crack model was used. A fracture mechanics approach employed in ATENA for softening behavior is based on the crack band model. Such a model substantially reduces mesh sensitivity [36]. A typical finite element model including the loading and support conditions of the test beams is presented in Figure 2.

The results of the nonlinear analysis strongly depend on the size of the finite mesh. Previous studies [36,37] have shown that the accuracy of numerical analysis results obtained by ATENA is sufficient using six finite elements per model height. According to the recommendations [36], the mesh size was normalized by assuming a 20 mm characteristic finite element length. Such normalization enables to eliminate the influence of the obtained results on the finite element mesh size. Isoparametric quadrilateral finite elements of eight degrees of freedom with four integration points were used to model the concrete beams. Reinforcement bars were modeled with truss finite elements. It should also be emphasized that shrinkage deformations prior to short-term loading have been taken into account in the numerical analysis. Shrinkage was modeled as a prescribed deformation affecting concrete macroelements [37]. The modeling aspects are described in more detail in reference [23].

## 3. Database of Experimental Results and Accuracy Analysis of Predictions

The database consists of data from 51 lightweight aggregate concrete flexural elements obtained from five different test programs reported by Carmo et al. [15], Sin et al. [14], Bernardo et al. [38], Wu et al. [39], and Vakhshouri [30]. The main characteristics of the flexural elements are given in Table 1 and Table 2. The reinforcement percentage of the experimental beams ranged from 0.33% to 2.82%, the density ranged from 1651 to 2000 kg/m^3^, and the compressive strength of concrete ranged from 20 to 70.1 MPa. Table 2 includes the characteristics of tensile strength of concrete and shrinkage deformations required for the numerical analysis. These characteristics were calculated according to the EC2 standard [32]. All the beams were tested under the four-point bending configuration (Figure 2) with the span and shear span parameters given in Table 1.

**Figure 3:**
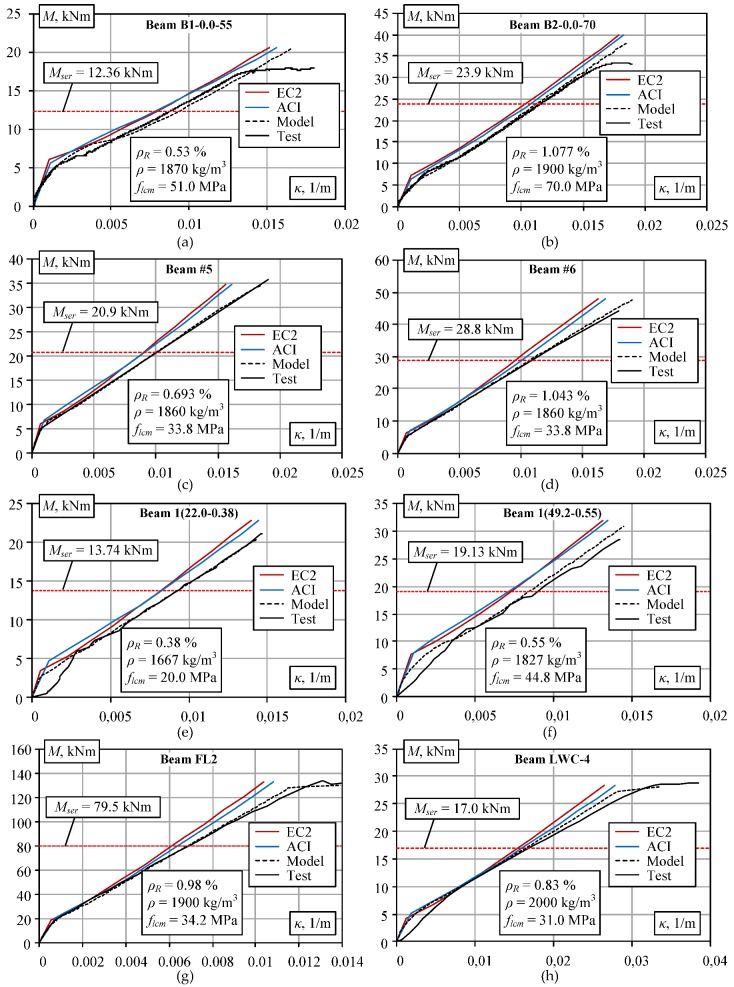
Comparison of theoretical and experimental moment-curvature diagrams: (**a**,**b**) Carmo et al. [15]; (**c**,**d**) Sin et al. [14]; (**e**,**f**) Bernardo et al. [38]; (**g**) Wu et al. [39]; and (**h**) Vakhshouri [30].

Comparison of the theoretical and experimental results of the selected eight beams is given in Figure 3. The theoretical analysis of the experimental beams was terminated at the load corresponding to the ultimate bending moment *M_Rm_* calculated according to EC2 [32]. In the figure, *ρ_R_* corresponds to the reinforcement percentage, *ρ* is the concrete density, and *f_lcm_* is the compressive strength of lightweight concrete. Results are compared at the service loading level taken as *M_ser_* = 0.6*M_Rm_* (shown in Figure 3 by red dashed line).

**Figure 4:**
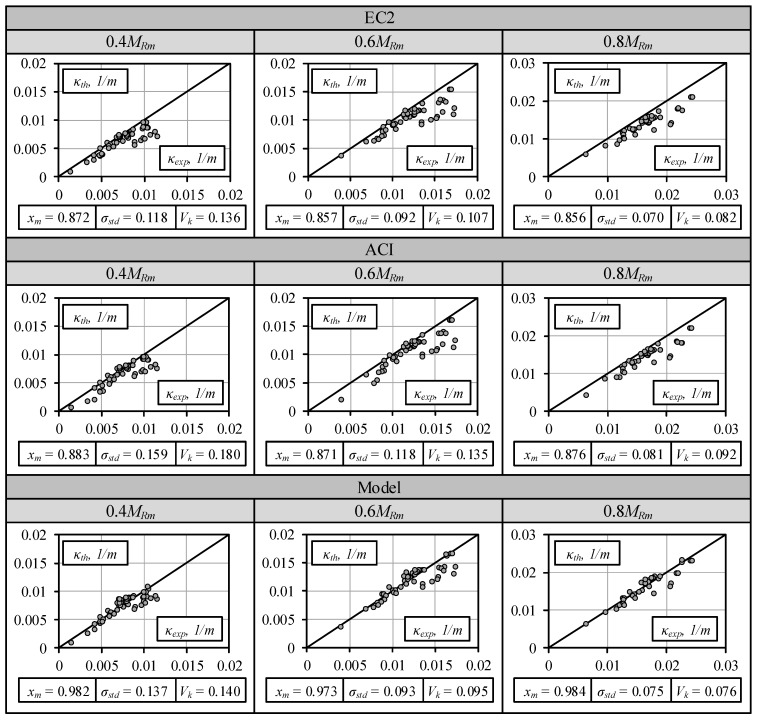
Comparison of experimental and theoretical curvatures at different load levels.

The comparison of the experimental curvatures against the theoretical results predicted by EC2 and ACI codes as well as the numerical approach is shown in Figure 4. The predictions were made for all the beams at three different loading levels: 0.4*M_Rm_*, 0.6*M_Rm_* (*M_ser_*), and 0.8*M_Rm_*. The mean value (*x_m_*), standard deviation (*σ_std_*), and coefficient of variation (*V_k_*) of the relative curvature (*κ_th_*/*κ_exp_*) are shown at the bottom of each graph in this figure. The mean errors of 12.8%, 14.3%, and 14.4% are obtained for the EC2 standard at load levels of 0.4*M_Rm_*, 0.6*M_Rm_*, and 0.8*M_Rm_*, respectively. The computational errors are rather modest and do not depend on the load level. Slightly smaller mean errors of 11.7%, 12.9%, and 12.4% assessed at the same load levels are obtained by using the ACI method. The predictions of the numerical model resulted in 1.8%, 2.7%, and 1.6% mean curvature errors obtained at load levels of 0.4*M_Rm_*, 0.6*M_Rm_*, and 0.8*M_Rm_*, respectively. It is important to note that EC2 and ACI code methods produced predictions that were too stiff.

**Figure 5:**
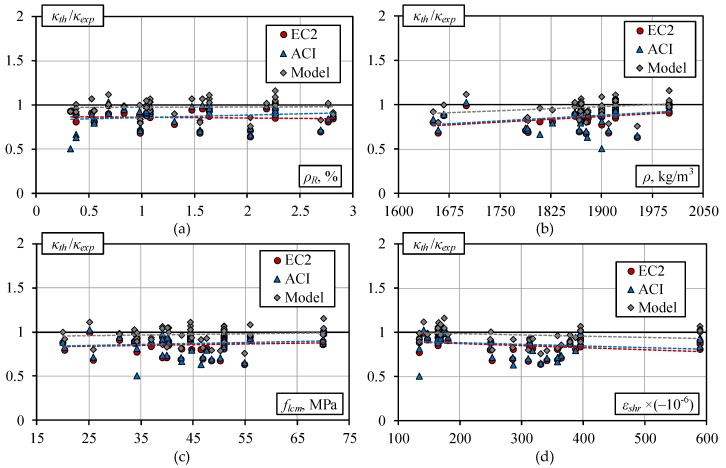
Relative curvatures estimate by different methods vs: (**a**) reinforcement ratio *ρ_R_*; (**b**) density *ρ*; (**c**) compressive strength *f_lcm_*; and (**d**) deformation of shrinkage *ε_shr_*.

Figure 5 shows the scatter of the normalized curvature predictions for ranges of material and geometrical parameters such as reinforcement percentage *ρ_R_*, concrete density *ρ*, compressive strength of concrete, *f_lcm_*, and shrinkage deformation *ε_shr_*. The latter results were obtained for all the beams at a service load *M_ser_* = 0.6*M_Rm_*. Figure 5 shows that none of the listed parameters, except concrete density and shrinkage strain, significantly affects the prediction accuracy in any of the methods. There is a general tendency that accuracy decreases with a rise in density and an increase in free shrinkage strain. Comparison of the results demonstrates that in all cases the proposed approach gives the most accurate predictions of mean normalized curvature as the respective trend-line approaches the unity line (shown as a black solid line).

## 4. Conclusions

A comparison analysis of theoretical and experimental results of deformations of reinforced lightweight concrete beams yields the following conclusions:

1. In engineering practice, the analysis of lightweight concrete elements is usually performed using the same algorithms used for normal concrete elements. The influence of lightweight concrete on the structural behavior is evaluated by additional density-dependent empirical coefficients. This prediction of the behavior of lightweight concrete often does not correspond to the real behavior of the structure. In many cases, the deformation of reinforced lightweight aggregate concrete elements is underestimated, and the resulting errors can reach 100%.
2. As an alternative to traditional engineering methods, nonlinear numerical algorithms based on physical material models that reflect the behavior of elements at various stages of operation may be used. Although many physical models of concrete have been proposed for the prediction of load carrying capacity and deformations in conventional reinforced concrete elements, there are no reliable physical models for the numerical analysis of reinforced lightweight aggregate concrete elements.
3. The constitutive model of cracked tensile lightweight concrete earlier proposed by the authors wasused in a comparative deformation analysis of reinforced lightweight concrete beams. Stresses characterizing the cracking limit were reduced in the modified model by considering the characteristics of formation of lightweight concrete cracks. The proposed model is approximated by a three-curve diagram. The rising part of the curve describes the elastic behavior of concrete before cracking. The horizontal and descending parts of the curve describe the stages of formation and development of cracks, respectively.
4. The adequacy of results obtained by design code techniques and numerical modeling method was verified by employing experimental data of reinforced lightweight aggregate concrete elements published in the literature. The data sample consisted of 51 flexural elements obtained from five different test programs. Numerical analysis of experimental beams was performed using the nonlinear finite element software ATENA and the constitutive model proposed by the authors to model the behavior of the cracked tensile concrete.
5. A comparison of theoretical and experimental results revealed that the most accurate calculation results were obtained by using the numerical model. At the service load level (*M_ser_* = 0.6*M_Rm_*, where *M_Rm_* is the theoretical average bearing bending moment calculated by EC2), the mean value of the relative curvature (*κ_th_*/*κ_exp_*) obtained by using the numerical model was 0.973, and the standard deviation was 0.093. By using the EC2 standard, the mean value of the relative curvature *κ_th_*/*κ_exp_* was 0.857, and the standard deviation was 0.092. The mean and standard deviation values of 0.871 and 0.118, respectively, were obtained by using the ACI standard method. The comparative analysis shows that EC2 and ACI code methods produced predictions that were too stiff.
6. The influence of the reinforcement percentage *ρ_R_*, concrete density *ρ*, compressive strength of concrete, *f_lcm_*, and shrinkage deformation *ε_shr_* has little effect on the mean curvatures predicted by the code and numerical methods. There is a general tendency that the accuracy of the methods decreases with the rise in density and the increase in free shrinkage strain. Comparison of results demonstrates that in all cases the proposed approach gives the most accurate predictions of mean normalized curvature.
7. In the future, the proposed constitutive model of lightweight aggregate concrete together with numerical finite element algorithms can be used as a reliable tool for improving the design code techniques or for adequate numerical modeling of reinforced lightweight aggregate concrete elements under short-term loading.

## Figures and Tables

**Table 1:** Main geometrical characteristics of experimental beams.

No.	Reference	Number of Beams	Span *L*_0_, m	Shear Span *a*, m	Depth *h*, mm	Width *b*, mm	Reinforcement Percentage *ρ_R_*, %
1	Carmo et al. [15]	13	2.80	1.0	270	120	0.53–2.82
2	Sin et al. [14]	18	2.80	1.0	300	150	0.69–2.27
3	Bernardo et al. [38]	14	2.40	0.8	300	150	0.38–2.69
4	Wu et al. [39]	3	4.00	1.4	400	250	0.33–1.310
5	Vakhshouri [30]	3	3.50	1.167	161	400	0.83
	Total:	51	2.40–4.00	0.8–1.4	161–400	120–400	0.33–2.82

**Table 2:** Main material characteristics of experimental beams.

No.	Reference	Concrete Density *ρ*, kg/m^3^	Compressive Strength *f_lcm_*, MPa	Tensile Strength *f_lctm_*, MPa	Shrinkage Strain *ε_shr_*, × −10^−6^
1	Carmo et al. [15]	1870–1900	37.0–70.0	2.84–4.37	313–395
2	Sin et al. [14]	1700–2000	25.1–70.1	1.72–4.17	141–175
3	Bernardo et al. [38]	1651–1953	20.0–55.0	1.36–3.78	249–388
4	Wu et al. [39]	1900	34.2	2.43	134
5	Vakhshouri [30]	2000	31.0	2.29	180
	Total:	1651–2000	20.0–70.1	1.36–4.37	134–395
